# A Global Study by ^1^H NMR Spectroscopy and SPME-GC/MS of the in Vitro Digestion of Virgin Flaxseed Oil Enriched or not with Mono-, Di- or Tri-Phenolic Derivatives. Antioxidant Efficiency of These Compounds

**DOI:** 10.3390/antiox9040312

**Published:** 2020-04-15

**Authors:** Jon Alberdi-Cedeño, María L. Ibargoitia, María D. Guillén

**Affiliations:** Food Technology, Faculty of Pharmacy, Lascaray Research Center, University of the Basque Country (UPV-EHU), Paseo de la Universidad nº 7, 01006 Vitoria-Gasteiz, Spain

**Keywords:** virgin flaxseed oil, in vitro digestion, oxidation, gamma-tocopherol, hydroxytyrosol acetate, dodecyl gallate, antioxidant efficiency, minor compounds, bioaccessibility

## Abstract

The effect of enriching virgin flaxseed oil with dodecyl gallate, hydroxytyrosol acetate or gamma-tocopherol on its in vitro digestion is studied by means of proton nuclear magnetic resonance and solid phase microextraction followed by gas chromatography/mass spectrometry. The extent and pattern of the lipolysis reached in each sample is analyzed, as is the bioaccessibility of the main oil components. None of the phenolic compounds provokes inhibition of the lipase activity and all of them reduce the lipid oxidation degree caused by the in vitro digestion and the bioaccessibility of oxidation compounds. The antioxidant efficiency of the three tested phenols is in line with the number of phenolic groups in its molecule, and is dose-dependent. The concentration of some minor oil components such as terpenes, sesquiterpenes, cycloartenol and 24-methylenecycloartenol is not modified by in vitro digestion. Contrarily, gamma-tocopherol shows very low in vitro bioaccessibility, probably due to its antioxidant behavior, although this increases with enrichment of the phenolic compounds. Oxidation is produced during in vitro digestion even in the presence of a high concentration of gamma-tocopherol, which remains bioaccessible after digestion in the enriched samples of this compound.

## 1. Introduction

Food lipid oxidation is an issue of great importance in field of the food technology because it causes food degradation with economic and health repercussions. This can occur during food processing and storage [1,2]. A common strategy to avoid oxidation consists of the incorporation of compounds with antioxidant abilities in foods. Many studies have been published on the incorporation of antioxidants in lipid foods, and have evaluated their antioxidant efficiency under different processing conditions [3,4,5].

In recent years, it has been shown that food lipid oxidation can also occur during digestion, and some studies have addressed the effect on digestion of the enrichment of lipids with compounds able to act as antioxidants. It should be noted that food oxidation during digestion can give rise to the formation of toxic compounds that can be directly absorbed. For this reason, the study of this issue could be considered even more important than the oxidation of food during processing or storage.

Among compounds having antioxidant abilities are phenolic compounds. These are usually secondary metabolites present in fruits and vegetables in very low concentrations; the healthy properties of such foods have been attributed to phenolic compounds. These compounds are considered to be free radical scavengers, and their antioxidant capacity has been related to the number and arrangement of their hydroxyl groups [6]. Due to their potential antioxidant abilities and to the beneficial health effects attributed to them [6,7], some studies have focused on the enrichment of lipid foods with different natural or synthetic phenolic compounds in order to prevent oxidation when they are submitted to thermal processes or to gastrointestinal digestion.

In this context, the object of the present research is to study the efficiency of different polyphenols such as epicatechin, resveratrol and caffeic and gallic acids in the inhibition of the oxidation of linoleic acid in authentic fluid from rat small intestine [8] by monitoring the oxidation levels according to the concentration of hexanal. The effect of some hydrophilic and lipophilic phenolic compounds on the oxidation efficacy of lipids during in vitro digestion of high- and low-fat beef meat was also studied by monitoring the concentration of malonaldehyde, 4-hydroxy-nonenal and hexanal [9]. Likewise, the antioxidant effect of 2,6-di-*tert*-butyl-hydroxytoluene (BHT) in the oxidation of cod liver oil during in vitro digestion was demonstrated [10]. Furthermore, several studies have analyzed the effect that enrichment of certain oils with alpha-tocopherol has on their oxidation when they are submitted to digestion conditions, with controversial results [9,11,12,13]. Finally, a comparative study of the effect of the enrichment of corn oil with alpha- and gamma-tocopherol showed that the former acts as prooxidant, which is in agreement with some of the aforementioned studies, whereas the second acts as antioxidant [14].

However, phenolic compounds, in addition to exhibiting antioxidant or prooxidant abilities, may also take part in other reactions during digestion because they come in contact with both all food components and with enzyme-containing digestive juices. It should be noted that phenolic compounds can react with proteins, and also with enzymes [15,16], reducing their activity and provoking negative effects on digestion by diminishing the extent of the hydrolytic reactions. In fact, it has been proven that tea polyphenols are able to inhibit the pancreatic lipase activity, thereby reducing the gastrointestinal lipolysis [17], and thus, the absorption of lipids. Likewise, it has been described that alkyl gallates are able to inhibit the activity of amylase, thereby reducing the absorption of carbohydrates [18,19].

All these studies suggest that the influence of phenolic compounds may not only decrease or increase oxidative reactions that can occur during lipid digestion, but also, in some cases, may affect the hydrolytic reactions that are the essence of the digestion process, which is to release absorbable building blocks.

Within this framework, this study examines the effect that the enrichment of virgin flaxseed oil with dodecyl gallate, hydroxytyrosol acetate or gamma-tocopherol has on its in vitro digestion process from a global perspective. This oil was selected because it can be considered a good model of omega-3 lipids due to its high concentration of linolenic groups; as such, it has a great tendency towards oxidation. The in vitro digestion model used will be a modified version of that developed by Versantvoort et al. (2005) [20], which has given satisfactory results in previous studies [21]. Samples before and after digestion will be studied by proton nuclear magnetic resonance spectroscopy (^1^H NMR) and solid phase microextraction followed by gas chromatography/mass spectrometry (SPME-GC/MS). Subjects such as hydrolysis degree, the occurrence or prevention of lipid oxidation and of the formation of oxidation compounds, and the in vitro bioaccessibility of the main components and of some minor virgin flaxseed oil components will be addressed. This study will shed light on the antioxidant efficiency of the aforementioned tri, di- and mono-phenols under in vitro digestion conditions, and determine whether these phenols are able to establish reactions with lipase, which would have a negative effect on the extent of lipolytic reactions.

## 2. Materials and Methods

### 2.1. Study Samples

The study was carried out with virgin flaxseed oil (F), purchased in a local supermarket. Its composition in molar percentages of linolenic (Ln), linoleic (L), oleic (O) and saturated (S) acyl groups was 55.7 ± 0.0%, 14.2 ± 0.3%, 20.5 ± 1.2% and 9.5 ± 0.9% respectively; this was determined from the ^1^H NMR spectral data as in previous studies [22,23]. In virgin flaxseed oil, terpenes and sesquiterpenes are abundant, as has been previously shown [24]; the oil used in this study was no exception, as will be shown later. Likewise, it is also known that the main sterols of this oil are cycloartenol and 24-methylenecyclartenol, and the main tocopherol is gamma-tocopherol [25,26]; the concentration of these compounds in this oil will be indicated later. The presence of these minor components is important because they have been recognized to display various biological activities [27,28,29,30].

Aliquots of virgin flaxseed oil, containing a natural concentration of gamma-tocopherol of 0.33 mmol/mol (AG + FA), were enriched separately in dodecyl gallate (DG) (purity 98%, from Alfa Aesar., GmbH & Co KG, Germany), hydroxytyrosol acetate (HTA) (purity of 99.54%, from Seprox Biotech, Madrid, Spain) and in gamma-tocopherol (γT) (purity ≥ 90%, Eisai Food & Chemical Co. Ltd., Tokyo, Japan). The samples enriched with dodecyl gallate were named FDG_1_ (with an enrichment of 0.14mmol DG/mol [FA + AG]_O_) and FDG_2_ (with an enrichment of 1.35mmol DG/mol [FA + AG]_O_). The samples enriched with hydroxytyrosol acetate were named FHTA_1_ (with an enrichment of 0.24mmol HTA/mol [FA + AG]_O_) and FHTA_2_ (with an enrichment of 2.65mmol HTA/mol [FA + AG]_O_). Finally, the samples enriched with different additional concentrations of gamma-tocopherol were named FγT_1_ (with an enrichment of 0.13mmol γT/mol [FA+AG]_O_), FγT_2_ (with an enrichment of 1.30mmol γT/mol [FA + AG]_O_), FγT_3_ (with an enrichment of 14.21mmol γT/mol [FA + AG]_O_) and FγT_4_ (with an enrichment of 32.79mmol γT/mol [FA + AG]_O_). These enrichment levels were set according to the solubility of these compounds in the oil. The above concentrations were obtained trying to reach enrichment degrees near 0.02 % and 0.2 % by weight for the three phenolic compounds and near 2% and 5% by weight in the case of gamma-tocopherol due to its high solubility in oils. However, these latter levels of enrichment were not possible for dodecyl gallate and hydroxytyrosol acetate because of their limited solubility.

### 2.2. Digestion Experiments

Aliquots (0.5 g) of the aforementioned samples were digested following the semistatic in vitro gastrointestinal digestion model developed by Versantvoort et al. (2005) [20]. This method was optimized to improve lipid digestion in an attempt to reach lipolysis levels of a similar order to in vivo digestion [21]. This method is a three-stage procedure to simulate digestive processes in the mouth, stomach and small intestine, by sequentially adding the corresponding digestive juices (saliva, gastric juice, duodenal juice and bile), whose compositions are given in Table S1 (see Supplementary Material). The first stage begins by adding 6 mL of saliva to the sample. After 5 min of incubation, 12 mL of gastric juice was added and the mixture was rotated at 40 rpm for 2 h at 37 ± 2 °C. One hour after the start of the gastric stage, the pH was set to between 2 and 3 with HCl (37%), simulating the gradual acidification of the chyme occurring in vivo. After 2 h of gastric stage, 2 mL of sodium bicarbonate solution (1 M), 12 mL of duodenal juice and 6 mL of bile juice were added. Subsequently, the pH was set to between 6 and 7, and the mixture was again rotated at 40 rpm and incubated at 37 ± 2 ℃ for 4 h. All the reagents and enzymes for the preparation of digestive juices were acquired from Sigma-Aldrich (St. Louis, MO, USA): α-amylase from *Aspergillus oryzae* (10,065, ~30 U/mg); pepsin from porcine gastric mucosa (P7125, ≥400 U/mg protein); amano lipase A from *Aspergillus niger* (534,781, ≥120,000 U/g); pancreatin from porcine pancreas (P1750); lipase type II crude from porcine pancreas (L3126, 100–500 U/mg protein (using olive oil, 30 min incubation) and bovine bile extract (B3883). The digested samples were named as the original samples preceded by D (DF, DFDG_1_, DFDG_2,_ DFHTA_1_, DFHTA_2_, DFγT_1_, DFγT_2_, DFγT_3_, and DFγT_4_). Three digestion experiments, each including duplicate samples, were performed. Blank samples corresponding to the mixture of juices submitted to digestive conditions were also taken for further analysis.

### 2.3. Digestate Lipid Extraction

Lipids of the digestates were extracted using dichloromethane as a solvent (CH_2_Cl_2_, HPLC grade, Sigma-Aldrich, ST. Louis, MO, USA) following a methodology that also allows fatty acids extraction, as in a previous studies [10]. This methodology involves a three-stage liquid–liquid extraction process with 20 mL of CH_2_Cl_2_ each. Afterwards, to ensure a complete protonation of fatty acids and/or the dissociation of the potential salts formed, the remaining water phase was acidified to pH 2 with HCl (37%) and a second extraction was carried out in three steps. All the CH_2_Cl_2_ extracts of each sample were mixed and the solvent was eliminated by means of a rotary evaporator under reduced pressure at room temperature in order to avoid lipid oxidation. The extraction yield was, in all cases, near 85%. These extracts contain triglycerides, diglycerides and monoglycerides, as well as fatty acids and tocopherols, and other minor lipophilic compounds either present in the original oil samples or formed from oil components in the digestion process.

### 2.4. Study by ^1^H NMR of Oil Samples and Lipid Extracts of Digestates

#### 2.4.1. Operating Conditions

The ^1^H NMR spectra of the original oil (F), of the oil samples enriched with the different compounds at the different concentrations (F, FDG_1_, FDG_2_, FHTA_1_, FHTA_2_, FγT_1_, FγT_2_, FγT_3_, and FγT_4_), and of the lipids extracted from their digestates (DF, DFDG_1_, DFDG_2,_ DFHTA_1_, DFHTA_2_, DFγT_1_, DFγT_2_, DFγT_3_, and DFγT_4_) were acquired in duplicate using a Bruker Avance 400 spectrometer operating at 400 MHz. For this purpose, the aforementioned samples (approximately 0.16 g) were dissolved in 400 µL of deuterated chloroform, which contained tetramethylsilane (TMS), as an internal reference (Cortec, Paris, France). First, a standard ^1^HNMR spectrum was acquired; then, in a second step, a NOESYGPPS experiment consisting of a one-dimensional ^1^H NMR pulse sequence with selective suppression of certain strong signals was carried out. This NOESYGPPS experiment allowed us to obtain a ^1^H NMR spectrum with higher sensitivity than the standard single pulse ^1^H NMR in the spectral region ranging from 5.8 to 9.8 ppm [31] at the cost of suppressing some signals in other regions. The relaxation and acquisition time used made it possible to completely relax the protons, thus making the signal areas proportional to the number of protons that generated them, allowing us to use them for quantitative purposes as in previous studies [32].

#### 2.4.2. Identification of the Components

**Figure 1:**
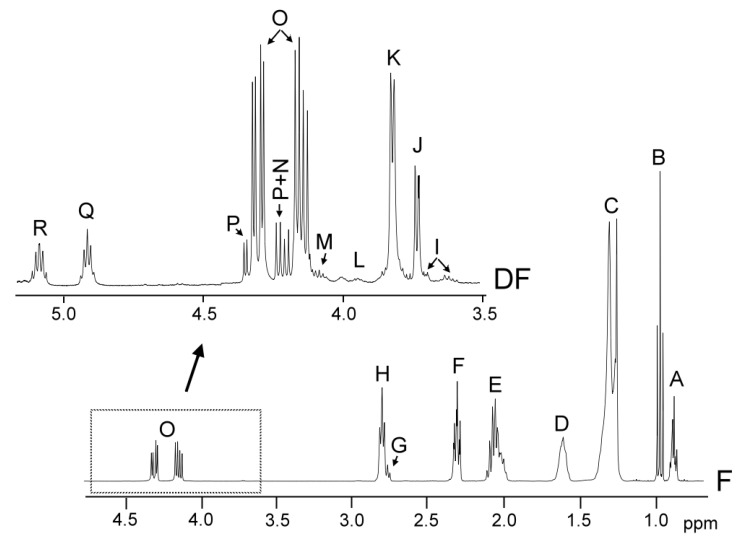
Region between 0.0 and 4.9 ppm of flaxseed virgin oil, F, ^1^H NMR spectrum, and enlargement of the region between 3.5 ppm and 5.10 ppm of the ^1^H NMR of the lipid extracted from the digestate, DF. The signal letters agree with those of Tables S2 and S3 of Supplementary Material.

The identification of the components present in the original oil, in the oil samples enriched with phenolic compounds, and in the lipid extracts of their digestates, was carried out on the basis of the assignments of the ^1^H NMR signals to the different kinds of hydrogen atoms, and, in short, to the different compounds. Figure 1 gives the spectral region between 0.0 and 4.9 ppm of the virgin flaxseed oil F ^1^H NMR spectrum and the region between 3.5 ppm and 5.10 ppm, conveniently enlarged, of the ^1^H NMR spectra of the lipids extracted of its digestate (DF), in which signals of the protons of their main components appear.

These signals, and other ones due to protons of oxidation compounds and minor components, are not shown in Figure 1, but are present in the spectra of all the samples here studied; their chemical shifts and assignments are given in Tables S2, S3, S4, and S5 (see Supplementary Material). Their assignments were made taking previous studies into account, as indicated in each table, or on the basis of the signals of the standard compounds acquired for this study. Among the latter are cycloartenol, hexanal and decanal, acquired from Sigma-Aldrich (St. Louis, MO, USA).

**Figure 2:**
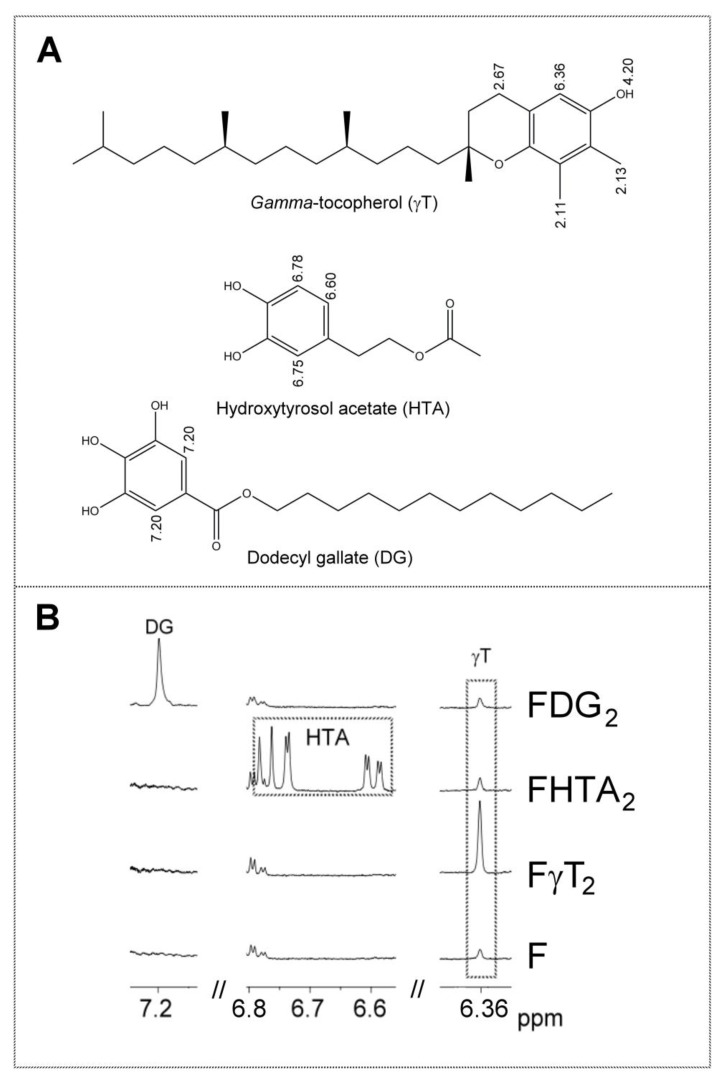
(**A**) Chemical structures of the phenolic compounds involved in this study (gamma-tocopherol γT, hydroxytyrosol acetate hydroxytyrosol acetate (HTA) and dodecyl gallate dodecyl gallate (DG), together with some chemical shifts (ppm) of the ^1^H NMR signals of some of their hydrogen atoms. (**B**) Enlargement of some regions of the ^1^H NMR spectra of nonenriched F and some enriched oil samples (FγT_2_, FHTA_2_ and FDG_2_), in which the signals of the aforementioned phenolic compounds appear. The signal letters agree with those of Table S5.

Table S2 shows the ^1^H NMR signals of the specific protons of the different glyceride structures, such as triglycerides, diglycerides and monoglycerides. Table S3 shows the ^1^H NMR signals of the protons of linolenic, linoleic, oleic, and saturated acyl groups and fatty acids, and those of methylenic protons supported on carbons atoms in alpha and beta position in relation to carbonyl-carboxyl groups. Table S4 shows the ^1^H NMR signals of protons of the oxidation compounds coming from the degradation of the main oil components which occurred during digestion. Finally, Table S5 gives the ^1^H NMR signals, present in Figure 2, of some protons of dodecyl gallate, of hydroxytyrosol acetate, and of gamma tocopherol and of free and esterified cycloartenol and 24-methylenecycloartenol. The areas of some of these spectral signals were used to quantify the concentrations of the different kinds of the aforementioned structures in the corresponding samples, as will be explained below.

#### 2.4.3. Quantifications from ^1^H NMR Spectral Data

This technique allows the estimation of the concentrations, expressed in different ways, of all of the aforementioned identified compounds. This is possible because, as explained, the area of the ^1^H NMR signals is proportional to the number of protons that generate the signal. The quantification of the different kinds of compounds or structures is explained below.

##### (A) Estimation of the Molar Percentage of the Different Kinds of Glycerides in the Digestates

Estimations of the molar percentages of each kind of glyceride structure can be carried out by using the intensity of the signals indicated in Tables S2 and S3, which are also shown in Figure 1. Although glycerol is formed during digestion, due to its polar nature, it is not present in the lipid extract of the digestate; however, its concentration can be estimated indirectly. This is possible because the concentrations of total fatty acids plus acyl groups, of only acyl groups, and of fatty acids released in the formation of diglycerides and monoglycerides can be determined from the ^1^H NMR data. Thus, the estimation of the molar percentage of triglycerides (TG), 1,2-diglycerides (1,2-DG), 1,3-diglycerides (1,3-DG), 2-monoglycerides (2-MG), 1-monoglycerides (1-MG), and glycerol (Gol) in relation to the total glyceryl structures present in the digestate was carried out using equations [Equations S1–S10] given in the Supplementary Material. They are based exclusively on the intensity of the ^1^H NMR spectral signals [33].

##### (B) Estimation of the Molar Percentage of Fatty Acids Plus Acyl Groups that Have Linolenic Structure in Relation to the Total of All Types of Fatty Acids and Acyl Groups in Digestates

In edible oils, the concentration of fatty acids is very small and, in many cases, inappreciable in comparison with the concentrations of acyl groups. However, during digestion, hydrolysis brings about the transformation of a certain number of acyl groups into fatty acids. The formed fatty acids maintain the same number of carbon atoms and unsaturation pattern as the starting acyl groups. Acyl groups and fatty acids having the same structure provide NMR spectra signals with a high degree of overlap that allows their joint quantification. In this study, the molar percentage of the linolenic acyl groups plus linolenic fatty acids in the digestates was estimated in relation to the total number of moles of all kinds of fatty acids plus acyl groups. This estimation was made using Equation S11, given in Supplementary Material, in which the areas of some signals that are shown in Figure 1 and in Table S3 are involved. This equation was employed in previous studies [22,23], but using the signal of methylenic protons supported on carbon atoms in alpha position in relation to carbonyl-carboxyl groups, instead of the signal of triglyceride protons used in edible oil studies.

##### (C) Estimation of the Concentration of Specific Compounds (SC) in Oil Samples and in the Digestates

The concentration of oxidation compounds, and of others, such as gamma-tocopherol, cycloartenol plus 24-methylenecycloartenol, dodecyl gallate, and hydroxytyrosol acetate, either in oils or digestates, can be estimated using the Equation S12, given in Supplementary Material, using the intensity of one of their nonoverlapped ^1^H NMR spectral signals, which are indicated in Figure 1 and Figure 2, and in Tables S3, S4, and S5. This equation allowed us to estimate the concentration of any compound in oils or in digestates in relation to the concentration of fatty acids plus acyl groups, which are considered the internal reference.

### 2.5. Study by SPME-GC/MS of the Headspace of Digestates and of the Mixture of the Digestive Juices Submitted to Digestion Conditions with the Virgin Flaxseed Oil

The extraction of the volatile components constituting the headspace of the several samples (0.5 g in a 10 mL screw-cap vial) was accomplished automatically using a CombiPAL autosampler (Agilent Technologies, Santa Clara, CA, USA). The samples studied were the several digestates (DF, DFDG_1_, DFDG_2,_ DFHTA_1_, DFHTA_2_, DFγT_1_, DFγT_2_, DFγT_3_, and DFγT_4_) and a mixture FDJ of digestive juices DJ, after undergoing digestion conditions, and virgin flaxseed oil F. The comparison of the headspaces of the several samples enabled us to deduce the changes brought about by in vitro digestion.

The fiber used for the extraction of the headspace components was coated with Divinylbenzene/Carboxen/Polydimethylsiloxane (DVB/CAR/PDMS, 50/30 μm film thickness, 1 cm long, and acquired from Supelco (Sigma-Aldrich, St. Louis, MO, USA)). It was inserted into the headspace of the sample and maintained for 55 min at 50 °C, after a pre-equilibration time of 5 min. The fiber containing the components extracted was desorbed for 10 min in the injection port (splitless mode with 5 min purge time) of a 7890A gas chromatograph equipped with a 5975C inert MSD with Triple Axis Detector (Agilent Technologies, Palo Alto, CA, USA) and a computer operating with the ChemStation program. A fused silica capillary column was used (60 m length, 0.25 mm internal diameter, 0.25 μm film thickness; from Agilent Technologies Inc., Palo Alto, CA), coated with a nonpolar stationary phase (HP-5MS, 5%phenyl methyl siloxane). The operating conditions were as follows: the injector and interface temperatures were held at 250 °C and 305 °C respectively, and helium at a constant pressure of 117 kPa (16.9 psi) was used as the carrier gas. The oven temperature was initially held at 50 °C for 5 min, increased from 50 °C to 300 °C at a rate of 4 °C/min, and then held at 300 °C for 30 min. Mass spectra were recorded at an ionization energy of 70 eV, with data acquisition in Scan mode. The temperatures of the ion source and the quadrupole mass analyzer were kept at 230 °C and 150 °C, respectively. A reference sample of known composition was periodically analyzed in order to verify the sensitivity of the SPME-GC/MS experiments, as in previous studies [34].

Identification of the headspace components was carried out using several commercial standard compounds acquired from Sigma-Aldrich (St. Louis, MO, USA). When standard compounds were not available, the identification was performed by matching the spectra obtained, higher than 85%, with those of commercial libraries (Wiley W9N08, Mass Spectral Database of the National Institute of Standards and Technology), or with spectra provided by the scientific literature, as in previous studies [34].

The semiquantification of the compounds was based on the area counts of the base peak (Bp) of the mass spectrum of each compound divided by 10^6^. When the Bp of a compound overlapped with an ion peak of the mass spectrum of another compound, an alternative ion peak was selected for the semiquantification of the former [34]. Although the chromatographic response factor of each compound is different, the area counts thus determined are useful for the comparison of the abundance of each compound in the different samples. The target compounds of this technique were terpenes and sesquiterpenes, which are characteristic minor volatile components of virgin flaxseed oil, and the volatile oxidation compounds formed in the in vitro digestion. The data given in the following tables are average values of triplicate experiments.

### 2.6. Statistical Analysis

The significance of the differences in the various kinds of data among samples was determined by one-way variance analysis (ANOVA) followed by Tukey *b* test at *p* < 0.05, using SPSS Statistics 24 software (IBM, NY, USA).

## 3. Results

As mentioned, the comparison between in vitro digestion of virgin flaxseed oil and that of the same enriched oil with different concentrations of dodecyl gallate, hydroxytyrosol acetate, or gamma-tocopherol was approached as globally as possible, attending to all aspects about which the applied techniques provide information.

### 3.1. Extent and Pattern of Lipolysis Produced by the in Vitro Digestion of Virgin Flaxseed Oil and Effect of the Enrichment with the Different Phenolic Compounds

In vitro digestion, as expected, caused the partial hydrolysis of the ester bond of triglycerides, yielding diglycerides, monoglycerides, fatty acids, and glycerol. The concentration of the different glyceryl species can be estimated from ^1^H NMR spectral data of the virgin flaxseed oil, F, and of the lipid extracts of the different digestates, as explained in the experimental section. The results obtained, indicated in Table 1, show that a very important percentage of triglycerides did not undergo hydrolysis but, in fact, remained as the main glyceride species after in vitro digestion. Monoglycerides and glycerol were present in the digestates in fairly high concentrations, indicating that the species able to be absorbed by enterocytes of the intestinal wall (monoglycerides and fatty acids) were present in significant concentrations after the in vitro digestion. Finally, diglycerides, which were not able to be absorbed were in the lowest concentration of all of the glyceride species.

As the statistical treatment shows, the enrichment of the oil with these phenolic compounds in the concentrations essayed did not significantly affect the hydrolysis observed in the digestion. This fact indicates that the added phenolic compounds did not inhibit the lipase activity, or in other words, did not react with lipases diminishing their activity, because, in this case, the hydrolysis yield should be smaller in the enriched samples than in the nonenriched sample. It should be mentioned that tea polyphenolic compounds, which can inhibit lipase, are very polar lipophobic compounds, some of which are even polymer polyphenols [17,35]. To the best of our knowledge, this is the first time that it has been shown that dodecyl gallate and hydroxytyrosol acetate are not able to inhibit lipase activity. The inability of gamma-tocopherol to inhibit lipase activity has been shown in a previous study and the results here obtained confirm this fact [14].

In contrast to this, and although the differences were not significant, a slightly smaller percentage of triglycerides, and a slightly higher percentage of glycerol in the digestates of the enriched samples than in the nonenriched sample was observed. This could suggest a slightly greater hydrolysis extent as a consequence of the enrichment in these phenolic compounds.

### 3.2. In Vitro Bioaccessibility of Oil Main Components and Influence of the Enrichment with Different Phenolic Compounds

The in vitro bioaccessibility of the main components of virgin flaxseed oil depends of both the extent and the pattern of lipolysis. As mentioned, of all the glyceryl species released during digestion, only fatty acids (FA) and monoglycerides (MG) are able to be absorbed by the intestinal wall. For this reason, the in vitro bioaccessibility of the oil main components is described by the ratio between the concentration of these absorbable species and all fatty acids (FA) plus acyl groups (AG) present in the corresponding digestate. This parameter is defined by the equation B_OMC_ = ([FA]+[MG])_D_ /([FA]+[AG])_D_, as in previous studies [14]. The data thus obtained are also given in Table 1. As can be observed, the in vitro bioaccessibility of the virgin flaxseed oil main components in the unenriched sample is only around fifty percent. Similar values were found for the virgin flaxseed oil samples enriched with the different phenolic compounds, without the differences among them being statistically significant. However, although these differences were not significant, in most cases, this parameter was slightly higher in the digestates of the oil samples enriched with the aforementioned phenolic compounds than in the digestate of the unenriched oil. This could be related to the antioxidant activity of the added compounds.

### 3.3. Oxidation Reactions during in Vitro Digestion of Virgin Flaxseed Oil and of Virgin Flaxseed Oil Samples Enriched in Dodecyl Gallate, Hydroxytyrosol Acetate and Gamma-Tocopherol

Oxidation reactions in oils lead to the degradation of some components and the formation of others which are new. For this reason, this subject can be tackled by either monitoring the changes in the concentration of the former as a consequence of the in vitro digestion, or by monitoring the formation of the latter after digestion, or by both.

#### 3.3.1. Changes Provoked by in Vitro Digestion, in the Concentration of Linolenic Structures. Antioxidant Efficiency of the Added Phenolic Compounds

In virgin flaxseed oil, linolenic acyl groups are the main ones, as indicated in the experimental section. Furthermore, the linolenic acyl group and linolenic fatty acid tend to oxidize more than any others due to their low oxidative stability. For this reason, if oxidation reactions take place during digestion, linolenic structures will be clearly affected, diminishing their concentrations in the sample. For this reason, they are the subject of this monitoring. ^1^H NMR spectroscopy allowed us to determine the concentration of linolenic structures in relation to the all fatty acids plus acyl groups in the sample subject of study. Table 2 gives the concentration of these structures in the oil before digestion and in the digestates of each of the samples. It can be observed that in vitro digestion of oil F causes a significant diminution in the concentration of linolenic structures. In other words, this group undergoes degradation which provokes a reduction in the molar percentage in relation to the total moles of all kinds of fatty acids plus acyl groups (AG+FA) from 55.7 % in F to 47.9% in DF.

The addition of different concentrations of phenolic compounds negates degradation during digestion to a certain extent, even at the lower enrichment level essayed. This indicates that the three compounds act as antioxidants. Nevertheless, their efficiency at avoiding the degradation of linolenic structures, or in other words, their oxidation, is not the same for the three antioxidants tested. From a comparison of data in Table 2, it is evident that in the range of enrichment levels studied, dodecyl gallate is the most efficient antioxidant of the three compounds. It can be observed that approximately half of the enrichment with dodecyl gallate is required (0.14 or 1.35 mmol/mol (AG + FA)) compared to that of hydroxytyrosol acetate (0.24 or 2.65 mmol/mol (AG + FA)) to reach the same reduction in the degradation of linolenic groups. Likewise, the higher antioxidant efficiency of dodecyl gallate than that of gamma-tocopherol is also evident because with similar enrichment levels of both compounds (see Table 2), the former avoids the degradation of linolenic groups to a greater extent than the latter. Finally, also taking data from Table 2, it is evident that hydroxytyrosol acetate is a more efficient antioxidant than gamma-tocopherol, because a concentration about five times greater of the latter is required to avoid a similar degree of oxidation.

These results demonstrate that during in vitro digestion of virgin flaxseed oil, the antioxidant efficiency of dodecyl gallate is greater than that of hydroxytyrosol acetate, which is, in turn, greater than that of gamma-tocopherol. This is also in agreement with the general idea that a greater number of phenolic groups in the molecule indicates a higher antioxidant ability, as described by some authors [6].

In the case of the enrichment with gamma-tocopherol and due to the availability of sufficient experimental data, it was possible to look for quantitative relationships between enrichment degree in the oil and linolenic structures concentration, expressed in molar percentage in relation to the total (AG+FA) moles in the corresponding sample, which is inversely related with oxidation level reached during in vitro digestion. It was found that the molar percentage of linolenic structures in the digestate [Ln] and enrichment level in gamma-tocopherol in the oil sample [γT], given in mmol/mol(AG + FA)_O_, fit the equation [Ln] = 51.06 + 1.16 Ln [γT], *R* = 0.9931, where Ln is the Napierian logarithm. This relationship is represented in Figure S1. According to this equation, the relationship between the molar percentage of linolenic structures, which is higher the higher the antioxidant efficiency, is directly related with gamma-tocopherol enrichment through the logarithmic relation given above. This means that an increase in the enrichment level of gamma-tocopherol when the concentration of this compound in the sample is low yields a higher antioxidant effect than the same increase in the enrichment level when the concentration of this compound in the oil sample is high. In other words, increased enrichment in gamma-tocopherol appreciably improves the antioxidant effect to a certain degree of enrichment, after which additional enrichments are much less efficient at improving this antioxidant effect (see Figure S1). According to this equation, to totally prevent linolenic oxidation during in vitro digestion requires an enrichment level of gamma-tocopherol near 54.59mmol/mol(AG + FA)_O_. This result indicates that the higher enrichment level essayed does not totally avoid oil oxidation during this in vitro digestion, as will be shown later.

The approach described above shows a methodology which may be used with any other antioxidant whenever enough experimental data are available. The generalization of its use will provide very interesting data, not only to estimate the antioxidant efficiency of different compounds, but also to predict, in a fairly accurate way, the enrichment degree required of a compound to avoid lipid oxidation during in vitro digestion.

#### 3.3.2. Formation of Oxidation Compounds Derived from Virgin Flaxseed Oil Main Components during in Vitro Digestion

As mentioned, the degradation of the oil main components during in vitro digestion gives rise to the formation of oxidation compounds, most of which should be present in the lipid extracts of the corresponding digestates. The detection and quantification of these can be carried out by means of ^1^H Nuclear Magnetic Resonance spectroscopy using both the standard and NOESYGPPS experiments, and by means of Solid Phase Microextraction (SPME) followed by Gas Chromatography Mass Spectrometry (GC/MS). The first technique views the sample as a whole and allows one to detect and quantify compounds of the same family having protons with similar electronic environments in a global way, without the previous steps of sample separation and without causing chemical changes in it. The second technique extracts the headspace components of the sample, among which, if the sample has undergone oxidation, there are volatile oxidation markers.

##### (a) Oxidation Compounds Detected in the Different Digestates by ^1^H NMR. Effect of the Enrichment in Phenolic Compounds

Two kinds of oxidation compounds were detected by this technique in the lipid extracts of the digestates, evidencing that oxidation took place during in vitro digestion. These are hydroperoxides supported on chains having *Z,E* conjugated dienic systems (HPO-c(*Z,E*)-dEs) derived from octadecatrienoic groups, which are primary oxidation compounds (multiplet signal at 6.58 ppm in the ^1^H NMR spectrum indicated in Table S4), and n-alkanals, which are secondary oxidation compounds (singlet at 9.75 ppm signal in the ^1^H NMR spectrum indicated also in Table S4). The concentrations of both kinds of compounds were determined using the aforementioned approach. They are given in Table 2 and are in agreement with the previous discussion relating to the degradation of linolenic structures. In general, the higher the degradation of linolenic structures, the higher the concentration of both kinds of oxidation compounds; likewise, the greater the enrichment in phenolic compounds in the sample, the lower the concentration of oxidation compounds in the digestates. Finally, it has again been demonstrated that, under in vitro digestion conditions, dodecyl gallate shows higher antioxidant efficiency than hydroxytyrosol acetate and gamma-tocopherol. To reduce the same oxidation level during in vitro digestion, measured by the concentration of hydroperoxides and n-alkanals, requires a level of enrichment with hydroxytyrosol acetate (or with gamma-tocopherol) that is approximately two times (or ten times) higher than with dodecyl gallate. Likewise, to reduce the same level of oxidation, measured by the aforementioned markers, requires a much higher concentration of gamma-tocopherol than of hydroxytyrosol acetate, which indicates that the efficiency of the latter as an antioxidant is higher than that of the former. Although hydroperoxides cannot be detected in the digestates of the more enriched in gamma-tocopherol samples, the presence of n-alkanals indicates that oxidation took place, which is in agreement with data of linolenic degradation and with data of volatile markers that will be discussed later.

As the rate of oxidation of fatty acids is much higher than that of the corresponding acyl groups, it could be thought that the first are lipid compounds that have been oxidized, incorporating hydroperoxy groups and conjugated dienic systems into their molecules, and also giving rise to the formation of aldehydes. This suggests that the newly-formed compounds could also be bioaccessible in the concentrations indicated in Table 2, i.e., the bioaccessibility of HPO-c(*Z,E*)-dEs (or of n-alkanals) will range between zero (or between 0.04mmol/mol(FA+AG)) in the digestates of the samples which are more enriched in gamma-tocopherol, and nearly 0.4 (or to near 0.09 mmol/mol(FA+AG)) in the unenriched sample or in the sample having the lowest enrichment in gamma-tocopherol. Furthermore, a smaller concentration of oxidation compounds in the enriched samples indicates that a lower level of degradation of fatty acids has taken place during digestion. This could be the reason why the samples enriched with phenolic compounds have slightly higher (although not statistically significant) bioaccessibility of the main components of virgin flaxseed oil than the unenriched sample.

##### (b) Oxidation Markers Detected by SPME-GC/MS in the Different Digestates. Effect of the Enrichment in Phenolic Compounds

The subjects of this study were, in addition to the digestates of the virgin flaxseed oil DF and those of the oil samples enriched with different phenols (i.e., DFDG_1_, DFDG_2_, DFHTA_1_, DFHTA_2_, DFγT_1_, DFγT_2_, DFγT_3_, and DFγT_4_), the mixture constituted by the juices submitted to in vitro digestion conditions and the undigested oil FDJ. The latter sample was taken as reference because it contains the oil not submitted to digestion, and for this reason, not to oxidation, but in a similar matrix to the digestates.

Among the main volatile compounds coming from lipid oxidation are aldehydes, furan derivatives, and ketones, and these are the target of this study. As an example, Figure S2 shows the region between 4–30 min of the total ion chromatogram obtained by SPME-GC/MS of the FDJ sample and of the DF digestate. In it, the increase in the intensity of some peaks or the appearance of new peaks related to the main volatile oxidation compounds formed during in vitro digestion can be observed. The abundances of the different compounds are directly related with the oxidation degree reached by the sample during digestion. Their values were estimated as indicated in the experimental section, and are given in Table 3, together with the enrichment levels of the different samples in the different phenols. Both sets of data allowed us to evaluate the antioxidant efficiency of each of these phenolic compounds in the oxidation which occurred in the virgin flaxseed oil during in vitro digestion.

It can be observed in Table 3 that the headspace of the sample FDJ, formed by the mixture of the undigested oil and the juices submitted to in vitro digestion conditions, contains a basal concentration of some of these oxidation markers, which are common in unoxidized oils. As shown, the in vitro digestion causes the oxidation of the main components of virgin flaxseed oil, generating volatile oxidation compounds derived mainly from the linolenic structures. For this reason, the volatile oxidation compounds are in much higher abundance in the headspace of DF sample than in the FDJ mixture (see Table 3). Found among them are typical compounds coming from the oxidation of linolenic structures, such as 2,4-heptadienals, 2,3-pentanedione, and 2,3-octanedione, as well as 3,5-octadien-2-one. However, the enrichment of the oil with phenolic compounds causes a reduction in the oxidation level reached during in vitro digestion. This is proved because, as can be observed in Table 3, the concentration of these volatile oxidation compounds in the headspace of the digestates of the samples enriched with phenolic compounds is smaller than that found in the headspace of the digestate of the unenriched sample. That is to say, the data in Table 3 confirm the facts inferred from the data coming from ^1^H NMR, not only regarding the oxidation level reached by the different samples, but also about the antioxidant efficiency of each phenolic compound.

In summary, under these in vitro digestion conditions, dodecyl gallate shows greater antioxidant efficiency than hydroxytyrosol acetate, which, in turn, has greater antioxidant efficiency than gamma-tocopherol. Nevertheless, it should be pointed out that the antioxidant efficiency order is not the same regarding the number of moles of the compound required to avoid or reduce oxidation, or if it refers to the amount (expressed in weight or in percentage in weight) of that compound required to reach the same endpoint. If antioxidant efficiency referred to the amount of compound, the hydroxytyrosol acetate would have higher antioxidant efficiency than dodecyl gallate, due to the great difference in the molecular weights of these compounds. For this reason, it is very important to indicate the units by which the antioxidant efficiency is measured. Perhaps a lack of clarity in this respect could be the cause of divergences between studies, regarding the antioxidant efficiency order of some compounds.

### 3.4. In Vitro Bioaccessibility of Some Minor Components of Virgin Flaxseed Oil, and Specifically, of Gamma-Tocopherol in the Different Digestates

As with other vegetable oils, this virgin flaxseed oil contains tocopherols and sterols. Furthermore, this oil also contains terpenes and sesquiterpenes, as has been shown in previous studies [24,26]. All these compounds have important biological activities, and in some cases, antioxidant abilities. Some of these minor oil components give signals in the ^1^H NMR spectra which do not overlap with any others, for which reason their concentrations in the sample before and after in vitro digestion can be estimated. These are (i) of the tocopherols, gamma-tocopherol (singlet signal at 6.36 ppm due to proton aromatic proton C-5, as indicated in Table S5 and shown in Figure 2); and (ii) of the sterols, cycloartenol and 24-methylenecycloartenol free or esterified (two doublet signals overlapping due to the methylene protons on carbon atom C-19 yielding a triplet joint signal centered at 0.33 ppm as is indicated in Table S5, which permits their joint quantification) [36]. They are respectively the main components of the tocopherols and sterols in this oil [26]. Other minor virgin flaxseed oil components such as terpenes and sesquiterpenes can, due to their volatility, be extracted from the headspace of the samples by means of solid phase microextraction (SPME), and can be separated, identified, and semiquantified by means of gas chromatography/mass spectrometry (GC/MS). For this reason, by using these techniques, the in vitro bioaccessibility of these compounds can be estimated.

As mentioned, the gamma-tocopherol concentration can be estimated in the different samples before and after in vitro digestion. The in vitro bioaccessibility of this compound can be expressed by its concentration in the digestate [γT]_D_, given in mmoles, in relation to the concentration of the main components also in the digestate [FA + AG]_D_, given in moles, by the equation B_γT_ = [γT]_D_/[FA + AG]_D_. This in vitro bioaccessibility definition gives information from a global, quantitative point of view. The values of the thus defined in vitro bioaccessibility of gamma-tocopherol are given in Table 4 for the different samples. It can be observed that during the in vitro digestion of the sample not enriched in phenols, almost the totality of the gamma-tocopherol present in the oil (0.33mmol/mol(FA + AG)) is degraded. In spite of this, oxidation of the sample took place. In the samples enriched in dodecyl gallate and hydroxytyrosol acetate, a larger amount of gamma-tocopherol remained undegraded than in the unenriched sample. This could indicate that these added di- or tri-phenolic compounds have some protective effect on gamma-tocopherol. It is noteworthy that these samples, in spite of the presence of gamma-tocopherol, were also oxidized, though to a lesser degree than the nonenriched sample. Finally, the amount of this compound that remains undegraded in the samples enriched in gamma-tocopherol after digestion increases according to the enrichment level, as would be expected. However, it was noticeable that even in the more gamma-tocopherol enriched samples, having important concentrations of this compound that did not degrade, a small oxidation degree took place, as shown before.

The in vitro bioaccessibility of gamma-tocopherol can also be expressed as the ratio between the concentration of the compound in the digestate [γT]_D_ and the concentration in the sample before digestion, [γT]_O_, as indicated by the equation B’_γT_= [γT]_D_/[γT]_O._ This parameter provides information about the degradation level of the gamma-tocopherol during the in vitro digestion. The values of the in vitro bioaccessibility thus defined are also given in Table 4. It can be observed in this table that in the samples enriched in dodecyl gallate and in hydroxytyrosol acetate, this parameter reaches values around four times higher than in the nonenriched sample, suggesting a potential protective effect of these phenolic compounds on gamma-tocopherol. Furthermore, in the samples enriched with the latter compound, its bioaccessibility increases, as does the enrichment level, and an important amount of the gamma-tocopherol remains after digestion which can then be absorbed, in agreement with BγT. This fact is important due to the biological activities attributed to this compound [27,28,29].

The concentration of cycloartenol and 24-methylenecycloartenol, free or esterified, as mentioned, can be estimated jointly in samples before and after digestion. It is noteworthy that its concentration remains unchanged during in vitro digestion, being of 0.6mmol/mol(FA + AG) in both undigested and digested samples. Their in vitro bioaccessibility, defined as the ratio between its concentrations in the undigested and digested samples, is the unity. This is an important fact because beneficial biological activities have been attributed to these compounds [30,37].

Likewise, an important group of terpenes and sesquiterpenes was present in the virgin flaxseed oil; their abundances before and after digestion, which reflect their concentrations in the samples, could be estimated by using SPME-GC/MS. An analysis of the results obtained indicated that the abundances of these compounds in the headspaces of the digestates of the samples enriched with different concentrations of each phenolic compound had very similar values; for this reason, they are given in Table 5 as an average value. Furthermore, it can also be observed in Table 5 that the abundance values of terpenes and sesquiterpenes in all digestates were very similar in both the unenriched and enriched in phenolic compounds samples; in fact, these abundances are even higher in the headspace of the digestates than in the FDJ mixture, probably due to the matrix effect. These results indicate that the concentrations of these compounds are not affected by either the digestion process or by the oxidation reactions that took place during this process. The preservation of these compounds during digestion, and by extension, their in vitro bioaccessibility to be absorbed, is of great interest, due to the biological activities attributed to them [38].

Finally, dodecyl gallate and hydroxytyrosol acetate have unoverlapped signals in the ^1^H NMR spectrum (see Table S5 and Figure 2). For this reason, if they are present in the lipid extract of the digestates, their spectra will show these signals. Signals of both compounds were visible in the spectra of the samples most enriched with these phenols, but they were not strong enough to be quantified due to their low intensity. This low concentration of phenols in the digestates could be due to either their being degraded almost totally during in vitro digestion, transforming them into other compounds by their action as antioxidants, or by other reactions among which their hydrolysis could be cited. In fact, some previous studies on hydroxytyrosol alkyl esters have described their partial hydrolysis under digestion conditions [39,40]. The hydrolysis of these compounds yields very polar compounds that will remain in the aqueous phase, so they are not detected in the lipid extract of the digestates. Although the hydrolysis of alkyl gallates in digestion has not been reported, to the best of our knowledge, it could not be discarded. Furthermore, reactions between phenolic compounds and the reactive aldehydes formed in the lipid oxidation during digestion could also take place [41], contributing to the disappearance of the phenolic compounds in the digestates.

## 4. Conclusions

The enrichment of virgin flaxseed oil with different concentrations of dodecyl gallate, hydroxytyrosol acetate, and gamma-tocopherol, does not appreciably modify either the lipolysis degree reached during in vitro digestion or its lipolysis pattern in comparison with nonenriched oil. These results show that the phenolic compounds involved in this study, under the in vitro digestion conditions tested, do not inhibit the activity of lipases, or in other words, do not react with them affecting the hydrolytic reactions. In contrast, and although no significant differences were observed, the digestates of the virgin flaxseed oil enriched in the aforementioned phenols showed, in general, a slightly smaller percentage of triglycerides, and a slightly higher percentage of glycerol than the digestate of the nonenriched sample. These results are also reflected in the bioaccessibility of the oil main components. In vitro digestion causes a small oxidation degree which clearly affects the linolenic structures whose concentrations diminish, forming hydroperoxides supporting conjugated *Z,E* dienic systems, which are primary oxidation compounds, and also volatile, secondary oxidation compounds, which are well known oxidation markers. Enrichment with the different phenols in the levels tested in this study reduced both the oxidation degree reached during the digestion of virgin flaxseed oil and the bioaccessibility of oxidation compounds, but not totally. Dodecyl gallate showed the best antioxidant efficiency, followed by hydroxytyrosol acetate and gamma-tocopherol. In the case of enrichment with gamma-tocopherol, it was observed that its antioxidant efficiency is related with the gamma-tocopherol concentration through a logarithmic relation. The concentrations of some minor components of the virgin flaxseed oil, such as cycloartenol, 24-methylenecycloartenol, terpenes, and sesquiterpenes, were not modified by in vitro digestion, showing an in vitro bioaccessibility near the unity. However, the in vitro bioaccessibility of the gamma-tocopherol contained in virgin flaxseed oil was very small, but increased in line with the enrichment of phenolic compounds. It has been shown that oxidation occurs during in vitro digestion, even in the presence of high concentrations of gamma-tocopherol which remain bioaccessible after digestion in the enriched samples in this compound. Finally, it should be the units by which the antioxidant efficiency was measured should be noted, since a lack of clarity in this respect could be the cause of the divergences among other studies regarding the antioxidant efficiency order.

## Figures and Tables

**Table 1:** Lipolysis extent. Molar percentages of triglycerides (TG), diglycerides (1,2-DG and 1,3-DG), monoglycerides (1-MG and 2-MG), and glycerol (Gol) in relation to the total glyceride structures in virgin flaxseed oil samples (F), in the digestate of this oil (DF), and in the samples enriched in dodecyl gallate, hydroxytyrosol acetate, and gamma-tocopherol (DFDG_1_, DFDG_2_, DFHTA_1_, DFHTA_2_ DFγT_1_, DFγT_2_, DFγT_3_ and DFγT_4_). Bioaccessibility of oil main components after in vitro digestion (B_OMC_), defined by the ratio (mol [FA] + [MG])_D_ /mol ([FA] + [AG])_D_.

Samples	Lipolysis Extent (Molar %)						Bioaccessibility (B_OMC_)
	TG (%)	1,2-DG (%)	2-MG (%)	1,3-DG (%)	1-MG (%)	Gol (%)	
Oil							
F	99.4 ± 0.0a	1.2 ± 0.0a	-	-	-	-	-
DF	33.1 ± 2.7b	18.1 ± 2.1b	21.7 ± 0.5a	4.8 ± 1.0a	2.2 ± 0.8a	20.2 ± 4.3a	0.52 ± 0.05a
Oil-dodecyl gallate							
DFDG_1_	33.6 ± 0.3b	16.8 ± 0.2b	20.3 ± 0.0a	6.0 ± 0.3a	1.6 ± 0.1a	21.9 ± 0.0a	0.51 ± 0.00a
DFDG_2_	32.7 ± 1.9b	17.3 ± 1.2b	20.6 ± 2.1a	4.7 ± 0.2a	2.6 ± 0.1a	22.1 ± 1.8a	0.53 ± 0.01a
Oil-hydroxytyrosol acetate							
DFHTA_1_	32.4 ± 0.5b	17.1 ± 0.4b	20.5 ± 1.5a	5.3 ± 0.2a	2.1 ± 0.1a	22.6 ± 2.0a	0.53 ± 0.01a
DFHTA_2_	31.2 ± 0.5b	16.6 ± 0.4b	22.3 ± 0.5a	4.3 ± 0.4a	2.0 ± 0.2a	23.7 ± 2.0a	0.55 ± 0.01a
Oil-gamma-tocopherol							
DFγT_1_	32.3 ± 0.9b	17.9 ± 2.5b	22.3 ± 0.7a	3.9 ± 0.9a	1.5 ± 0.1a	22.2 ± 3.3a	0.53 ± 0.01a
DFγT_2_	32.4 ± 2.0b	16.9 ± 0.1b	20.3 ± 0.5a	5.5 ± 2.9a	1.4 ± 0.1a	23.4 ± 0.5a	0.53 ± 0.00a
DFγT_3_	30.1 ± 0.7b	17.3 ± 0.0b	20.2 ± 0.6a	7.4 ± 0.4a	2.2 ± 0.1a	22.7 ± 0.9a	0.53 ± 0.00a
DFγT_4_	32.4 ± 0.0b	17.6 ± 0.9b	20.6 ± 0.6a	4.4 ± 0.5a	2.1 ± 0.0a	22.9 ± 0.9a	0.53 ± 0.01a

Different letters within each column indicate a statistically significant difference among the samples (*p* < 0.05). A dash indicates “not detected”.

**Table 2:** Concentration of linolenic structures given in molar percentage in relation to the total moles of AG + FA present in the virgin flaxseed oil F, in the digestate of this oil DF, and in those of the samples enriched in hydroxytyrosol acetate, dodecyl gallate, and gamma-tocopherol (DFDG_1_, DFDG_2_, DFHTA_1_, DFHTA_2_, DFγT_1_, DFγT_2_, DFγT_3_, and DFγT_4_), together with the enrichment level of phenolic compounds in the samples before digestion. The concentrations of oxidation compounds such as hydroperoxides and n-alkanals in the same samples determined from ^1^H NMR spectral data.

Oil, Digestates, and Enrichment Level in Phenolic Compounds of the Oil Samples before Digestion, Given in mmol/mol [AG + FA]	Linolenic Structures (%)	Oxidation Compounds	
		HPO-c(*Z,E*)-dEs mmol/mol [AG + FA]	n-alkanals mmol/mol [AG + FA]
Oil			
F	55.7 ± 0.0a	-	-
DF	47.9 ± 0.8d	0.39 ± 0.04a	0.09 ± 0.00a
Oil-dodecyl gallate			
DFDG_1_ (0.14)	50.9 ± 1.1abc	0.33 ± 0.04ab	0.08 ± 0.01a
DFDG_2_ (1.35)	53.1 ± 1.8ab	0.28 ± 0.02b	0.07 ± 0.01a
Oil-hydroxytyrosol acetate			
DFHTA_1_ (0.24)	50.9 ± 1.1abc	0.30 ± 0.02b	0.08 ± 0.00a
DFHTA_2_ (2.65)	53.7 ± 0.3ab	n.d.	0.07 ± 0.00a
Oil-gamma-tocopherol			
DFγT_1_ (0.13)	48.5 ± 3.1d	0.40 ± 0.02a	0.09 ± 0.01a
DFγT_2_ (1.30)	51.8 ± 1.0abc	0.33 ± 0.02ab	0.07 ± 0.01a
DFγT_3_ (14.21)	53.8 ± 0.1ab	-	0.07 ± 0.00a
DFγT_4_ (32.79)	55.2 ± 0.2a	-	0.04 ± 0.00b

Different letters within each column indicate a statistically significant difference among the samples (*p* < 0.05), -: not detected; n.d.: not determined due to interfering signals; HPO-c(*Z,E*)-dEs: hydroperoxy-conjugated (*Z,E*)-dienes.

**Table 3:** Abundances of some volatile oxidation markers extracted by SPME from the headspace of the mixture of digestive juices and virgin flaxseed oil FDJ, from the digestate of this oil DF, and from the digestates of the samples enriched in dodecyl gallate, hydroxytyrosol acetate, and gamma-tocopherol (DF, DFDG_1_, DFDG_2_, DFHTA_1_, DFHTA_2_, DFγT_1_, DFγT_2_, DFγT_3_, and DFγT_4_), separated, identified, and semiquantified by GC/MS, together with the enrichment level of each phenol in each oil sample before digestion given in mmol/mol (AG + FA)_O_. Data are expressed as area counts of the mass spectra base peak (Bp) of each compound multiplied by 10^−6^, obtained as the average of two determinations, together with their standard deviations.

Compound (Molecular Weight)	Bp	FDJ (0.0)	DF (0.0)	DFDG_1_ (0.14)	DFDG_2_ (1.35)	DFHTA_1_ (0.24)	DFHTA_2_ (2.65)	DFγT_1_ (0.13)	DFγT_2_ (1.30)	DFγT_3_ (14.21)	DFγT_4_ (32.79)
Aldehydes											
*Alkanals*											
Pentanal (86) *	44	10.60 ± 3.78	65.99 ± 5.57	38.23 ± 4.68	35.09 ± 1.29	54.45 ± 0.84	35.19 ± 0.68	77.53 ± 7.47	55.66 ± 3.44	39.70 ± 4.33	31.03 ± 2.10
Hexanal (100) *	44	7.85 ± 2.16	63.22 ± 9.92	56.89 ± 7.03	52.86 ± 1.25	66.69 ± 6.45	51.03 ± 5.29	63.71 ± 2.84	55.52 ± 3.62	45.84 ± 2.07	39.02 ± 0.55
Heptanal (114) *	70	0.49 ± 0.05	4.10 ± 0.03	3.30 ± 0.98	2.16 ± 0.23	3.61 ± 0.35	3.70 ± 0.05	4.67 ± 0.07	4.17 ± 0.18	3.14 ± 0.01	2.80 ± 0.05
Octanal (128) *	41	-	7.71 ± 0.51	-	-	-	-	8.36 ± 0.20	-	-	-
Nonanal (142) *	57	2.97 ± 0.80	12.05 ± 1.17	15.73 ± 4.14	10.02 ± 0.57	11.21 ± 3.46	10.75 ± 0.32	14.98 ± 4.65	11.79 ± 0.55	9.55 ± 0.67	8.70 ± 0.12
*Total*		21.91 ± 6.78	153.07 ± 17.52	114.14 ± 16.83	100.14 ± 3.34	135.96 ± 11.10	100.67 ± 6.34	169.25 ± 15.23	127.14 ± 7.79	98.23 ± 7.09	81.55 ± 2.82
*(E)-2-Alkenals*											
(*E*)-2-Pentenal (84)	55	2.23 ± 0.29	10.67 ± 0.34	5.24 ± 0.44	5.64 ± 0.13	6.70 ± 1.15	5.12 ± 1.15	8.25 ± 0.82	5.41 ± 0.55	5.96 ± 1.04	5.46 ± 0.29
(*E*)-2-Hexenal (98) *	41	0.39 ± 0.07	1.38 ± 0.03	0.77 ± 0.07	0.43 ± 0.15	0.79 ± 0.03	0.60 ± 0.01	1.03 ± 0.03	1.14 ± 0.16	0.95 ± 0.03	0.53 ± 0.03
(*Z*)-4-Heptenal (112)	41	1.53 ± 0.06	6.09 ± 1.40	4.27 ± 0.49	4.55 ± 0.44	4.78 ± 0.06	3.74 ± 0.42	6.18 ± 1.82	4.50 ± 0.38	3.61 ± 0.44	2.87 ± 0.14
(*E*)-2-Nonenal (140) *	55	-	0.32 ± 0.01	-	-	-	-	2.9 ± 0.1	-	-	-
*Total*		4.15 ± 0.41	18.46 ± 1.77	10.21 ± 1.00	10.63 ± 0.73	12.26 ± 1.24	10.02 ± 1.82	15.76 ± 2.69	11.05 ± 1.09	10.52 ± 1.58	8.86 ± 0.46
*2,4-Alkadienals*											
(*E,E*)-2,4-Hexadienal (96) *	81	-	2.86 ± 0.21	1.19 ± 0.11	1.14 ± 1.8	1.60 ± 0.16	1.18 ± 0.34	1.64 ± 0.04	1.87 ± 0.04	1.25 ± 0.14	1.06 ± 0.00
(*Z,E*)-2,4-Heptadienal (110)	81	3.31 ± 0.93	19.66 ± 0.62	9.35 ± 0.15	7.97 ± 0.37	14.02 ± 0.50	8.07 ± 1.48	14.05 ± 0.46	12.48 ± 0.08	11.54 ± 1.35	8.35 ± 0.78
(*E,E*)-2,4-Heptadienal (110) *	81	2.24 ± 0.39	21.30 ± 0.66	17.38 ± 0.66	10.49 ± 0.19	19.02 ± 0.08	11.62 ± 0.66	19.79 ± 3.60	13.34 ± 0.29	12.77 ± 1.19	11.21 ± 0.44
*Total*		5.55 ± 1.32	43.81 ± 1.48	27.92 ± 0.92	19.60 ± 0.74	34.64 ± 0.73	20.87 ± 2.49	35.48 ± 4.12	27.69 ± 0.41	25.57 ± 2.68	20.63 ± 1.23
Aromatic aldehydes											
Benzaldehyde (106) *	106	3.85 ± 1.71	8.15 ± 1.06	4.50 ± 0.30	5.11 ± 0.31	6.79 ± 0.59	6.71 ± 0.89	7.08 ± 1.01	6.99 ± 1.03	6.32 ± 0.51	5.25 ± 0.13
Furan derivatives											
Furan, 2-ethyl (96) *	81	1.88 ± 0.03	6.29 ± 2.64	4.51 ± 0.64	3.59 ± 0.22	4.64 ± 0.38	3.12 ± 0.43	6.91 ± 2.0	3.30 ± 0.72	3.34 ± 0.89	2.81 ± 0.77
Furan, 2-butyl (124)	81	0.29 ± 0.04	0.46 ± 0.08	-	-	-	-	0.70 ± 0.28	0.70 ± 0.01	-	-
Furan, 2-pentyl (138) *	81	4.20 ± 1.11	11.69 ± 0.71	9.83 ± 0.43	8.50 ± 0.42	11.20 ± 0.37	10.13 ± 0.13	11.12 ± 0.50	11.53 ± 0.47	11.28 ± 0.09	9.25 ± 0.18
*Total*		6.37 ± 1.18	18.43 ± 3.42	13.94 ± 1.04	12.09 ± 0.64	15.84 ± 0.75	13.25 ± 0.56	18.72 ± 0.98	15.52 ± 1.20	14.62 ± 0.98	12.06 ± 0.95
Ketones											
2,3-Pentanedione (100) *	43	1.64 ± 0.12	26.39 ± 8.58	24.25 ± 2.21	9.78 ± 0.30	19.24 ± 0.77	16.58 ± 0.43	25.22 ± 5.44	14.00 ± 1.73	4.54 ± 0.05	3.32 ± 0.41
2-Hexanone (100)	43	0.58 ± 0.02	2.46 ± 0.10	1.20 ± 0.28	1.55 ± 0.43	1.83 ± 0.04	1.02 ± 0.03	1.94 ± 0.15	1.38 ± 0.06	1.59 ± 0.00	1.18 ± 0.21
2-Heptanone (114) *	43	5.63 ± 0.12	9.27 ± 0.56	9.58 ± 1.01	6.75 ± 0.14	8.68 ± 1.65	7.40 ± 0.12	9.79 ± 1.37	8.24 ± 0.54	7.31 ± 0.83	6.79 ± 0.42
2,3-Octanedione (142)	43	1.49 ± 0.29	6.92 ± 1.25	6.09 ± 0.13	4.38 ± 0.00	6.68 ± 0.49	5.50 ± 0.31	6.50 ± 0.46	6.31 ± 0.34	4.55 ± 0.14	2.21 ± 0.01
2-Octanone (128) *	43	2.14 ± 0.01	3.84 ± 0.57	3.71 ± 0.70	3.26 ± 0.07	3.76 ± 0.08	2.56 ± 0.34	4.24 ± 0.47	3.99 ± 0.24	3.67 ± 0.14	2.47 ± 0.05
3-Octen-2-one (126)	55	1.15 ± 0.12	1.91 ± 0.37	1.52 ± 0.04	1.21 ± 0.21	2.00 ± 0.11	0.96 ± 0.05	1.85 ± 0.34	1.95 ± 0.29	1.05 ± 0.48	0.96 ± 0.32
3*E*,5*Z*-Octadien-2-one (124) *	95	0.78 ± 0.08	9.40 ± 0.18	5.40 ± 0.11	2.17 ± 0.01	7.98 ± 0.05	2.70 ± 0.19	6.13 ± 0.16	3.23 ± 0.11	2.15 ± 0.07	1.97 ± 0.13
*Total*		13.41 ± 0.69	60.18 ± 11.62	49.94 ± 4.49	29.09 ± 1.16	50.16 ± 3.20	36.72 ± 1.41	55.67 ± 8.39	39.11 ± 3.32	24.85 ± 1.72	18.90 ± 1.56
Alcohols											
1-Hexanol (102) *	56	10.53 ± 1.15	12.55 ± 1.51	13.15 ± 0.16	9.02 ± 0.45	9.38 ± 0.52	8.61 ± 0.02	12.09 ± 0.09	9.73 ± 0.85	8.02 ± 0.73	5.26 ± 0.19
1-Octen-3-ol (128) *	57	-	8.62 ± 0.97	11.09 ± 0.19	6.72 ± 0.56	10.74 ± 0.87	6.10 ± 0.53	9.63 ± 0.28	6.24 ± 0.57	5.51 ± 0.35	2.53 ± 0.11
*Total*		10.53 ± 1.15	21.17 ± 2.48	24.24 ± 0.35	15.74 ± 1.01	20.12 ± 1.38	14.72 ± 0.55	21.72 ± 0.37	15.97 ± 1.43	13.53 ± 1.09	7.79 ± 0.29

* Asterisked compounds were acquired commercially and used as standards for identification purposes; -: not detected.

**Table 4:** In vitro bioaccessibility of gamma-tocopherol in the different samples, defined by B_γT_ = mmol (γT)_D_/mol (AF + GA)_D_ and by B’_γT_ = mmol(γT)_D_/mmol (γT)_O_ Values are the average of two determinations together with their standard deviations.

Samples	B_γT_	B’_γT_
Oil		
DF	0.04 ± 0.01	0.12 ± 0.00
Oil-dodecyl gallate		
DFDG_1_	0.14 ± 0.00	0.42 ± 0.01
DFDG_2_	0.16 ± 0.01	0.48 ± 0.03
Oil-hydroxytyrosol acetate		
DFHTA_1_	0.13 ± 0.00	0.39 ± 0.02
DFHTA_2_	0.16 ± 0.01	0.48 ± 0.01
Oil-gamma-tocopherol		
DFγT_1_	0.09 ± 0.01	0.19 ± 0.05
DFγT_2_	0.95 ± 0.03	0.58 ± 0.02
DFγT_3_	11.41 ± 0.29	0.78 ± 0.02
DFγT_4_	28.73 ± 0.27	0.86 ± 0.01

**Table 5:** Terpenes and sesquiterpenes of virgin flaxseed oil, detected by SPME-GC/MS in the headspaces of the mixture of digestive juices submitted to digestive conditions and virgin flaxseed oil FDJ, of the digestate of this oil DF and of the digestates of the samples enriched with different levels of dodecyl gallate, hydroxytyrosol acetate, and gamma-tocopherol (DFDG, DFHTA, DFγT). Data are average abundances expressed as area counts of the mass spectra base peak Bp of each compound multiplied by 10^−6^, together with their standard deviations. For samples enriched with phenolic compounds, data given are average values of the abundances of the headspace of digestates coming from samples having different enrichment levels of phenolic compounds.

*Terpenes* *and Sesquiterpenes*	Bp	FDJ	DF	DFDG_average_	DFHTA_average_	DFγT_average_
alpha-thujene	93	24.33 ± 2.42a	41.87 ± 5.39b	38.49 ± 6.62b	38.30 ± 1.85b	44.45 ± 3.34b
alpha-pinene *	93	18.81 ± 2.07a	35.15 ± 8.52b	31.32 ± 4.24b	30.79 ± 1.48b	33.11 ± 4.63b
Sabinene	93	2.10 ± 0.13a	3.27 ± 0.01b	2.44 ± 0.42a	2.49 ± 0.08a	2.80 ± 0.41ab
beta-pinene *	93	4.70 ± 0.25a	10.90 ± 0.74b	8.88 ± 1.34bc	8.15 ± 0.31c	8.86 ± 1.09bc
l-phellandrene *	93	0.28 ± 0.07a	0.45 ± 0.08b	0.50 ± 0.07b	0.39 ± 0.06ab	0.49 ± 0.06b
delta-3-carene	93	1.04 ± 0.15a	2.79 ± 0.07b	2.40 ± 0.11bc	1.92 ± 0.09c	2.30 ± 0.28c
alpha-terpinene *	93	0.56 ± 0.28a	1.70 ± 0.02b	1.22 ± 0.18cd	1.14 ± 0.15d	1.56 ± 0.15bc
Cymene *	119	189.10 ± 0.54a	240.24 ± 24.37b	243.91 ± 15.58b	268.78 ± 12.34b	248.03 ± 18.38b
Limonene *	68	18.32 ± 1.29a	35.81 ± 0.95b	37.24 ± 1.54b	32.21 ± 1.88b	33.96 ± 3.74b
gamma-terpinene *	93	2.45 ± 0.31a	4.95 ± 0.58b	4.10 ± 0.78b	4.27 ± 0.12b	4.65 ± 0.49b
alpha-terpinolene *	93	0.25 ± 0.01a	0.35 ± 0.07ab	0.45 ± 0.06b	0.40 ± 0.06ab	0.43 ± 0.08b
4-Terpineol *	71	0.22 ± 0.03a	2.49 ± 0.04b	2.15 ± 0.52bc	1.77 ± 0.05c	2.55 ± 0.19b
alpha-terpineol *	59	0.28 ± 0.03a	1.61 ± 0.13b	1.27 ± 0.22b	1.21 ± 0.08b	1.59 ± 0.18b
Carvone *	82	1.63 ± 0.30a	4.36 ± 0.46bc	4.54 ± 0.40c	3.38 ± 0.25b	4.75 ± 0.62c
alpha-copaene	119	0.21 ± 0.03a	0.54 ± 0.02bc	0.46 ± 0.15bc	0.40 ± 0.02b	0.63 ± 0.04c
beta-elemene	93	0.10 ± 0.02a	0.26 ± 0.0b	0.25 ± 0.04b	0.20 ± 0.01b	0.26 ± 0.02b
Calarene	161	0.30 ± 0.01a	0.80 ± 0.04bc	0.74 ± 0.23bc	0.65 ± 0.04b	0.97 ± 0.11c
Cadinene	161	0.10 ± 0.03a	0.32 ± 0.04bc	0.27 ± 0.07bc	0.24 ± 0.02b	0.35 ± 0.04c

Different letters within each row indicate a statistically significant difference among the samples (*p* < 0.05). * Asterisked compounds were acquired commercially and used as standards for identification purposes.

## Supplementary Materials

The following are available online at https://www.mdpi.com/2076-3921/9/4/312/s1, Table S1: Composition and pH values of the juices employed in the in vitro digestion model employed in this study; Table S2: Chemical shift assignments and multiplicities of the ^1^H NMR signals in CDCl_3_ of protons of glycerides; Table S3: Chemical shift assignments and multiplicities of the ^1^H NMR signals in CDCl_3_ of protons of acyl groups and fatty acids; Table S4: Chemical shift assignments and multiplicities of the ^1^H NMR signals in CDCl_3_ of protons of some oxidation compounds detected in the digestates and formed during the in vitro digestion; Table S5: Chemical shift assignments and multiplicities of the ^1^H NMR signals in CDCl_3_ of protons of cycloartenol and methylencycloartenol, esters of cycloartenol and methylencycloartenol, gamma-tocopherols, hydroxytyrosol acetate and dodecyl gallate detected in the samples before and after in vitro digestion; Equations used for the quantification from ^1^H NMR spectral data of several compounds present in the starting samples and/or in the lipid extract of the digestates; Figure S1. Graphical representation of linolenic structures concentration in the digestates of the different virgin flaxseed oil samples enriched in gamma-tocopherol given in molar percentage of [Ln] referred to the total moles of [AG + FA]_D_ versus enrichment level of gamma-tocopherol in the corresponding oil samples, given in mmol γT/mol[AG + FA]_O_. Figure S2. Region between 4–30 min of the total ion chromatogram obtained by SPME-GC/MS of the FDJ sample and of the digestate of the virgin flaxseed oil DF.
