# Supplementary material for: A Global Study by 1H NMR Spectroscopy and SPME-GC/MS of the in Vitro Digestion of Virgin Flaxseed Oil Enriched or not with Mono-, Di- or Tri-Phenolic Derivatives. Antioxidant Efficiency of These Compounds
Source: Antioxidants (Basel). 2020 Apr 15;9(4):312. doi: 10.3390/antiox9040312 (PMC7222186; doi:10.3390/antiox9040312)
Supplement: Supplementary file 1 [file antioxidants-09-00312-s001.pdf]

SUPPLEMENTARY MATERIAL

A global study by ^{1}H NMR spectroscopy and SPME-GC/MS of the *in vitro* digestion of virgin flaxseed oil enriched or not with mono-, di- or tri-phenolic derivatives. Antioxidant efficiency of these compounds

J. Alberdi-Cedeño, María L. Ibargoitia, María D. Guillén*

Food Technology. Faculty of Pharmacy. Lascaray Research Center. University of the Basque Country (UPV-EHU). Paseo de la Universidad nº 7, 01006 Vitoria-Gasteiz, Spain, Telf: 34-945-013081, Fax: 34-945-013014.

E-mail: mariadolores.guillen@ehu.es*

Table S1. Composition and pH values of the juices employed in the *in vitro* digestion model employed in this study.

**Components**	**Saliva**	**Gastric juice**	**Duodenal juice**	**Bile juice**
KCl (mmol/L)	12.02	11.06	7.57	5.05
NaCl (mmol/L)	5.10	47.09	119.98	89.99
$NaHCO_3$ (mmol/L)	20.17	-	40.33	68.86
NaH_2PO_4 (mmol/L)	7.40	0.22	-	-
NH_4Cl (mmol/L)	-	5.72	-	-
KH_2PO_4 (mmol/L)	-	-	0.59	-
Na_2SO_4 (mmol/L)	4.79	-	-	-
KSCN (mmol/L)	2.06	-	-	-
$MgCl_2$ (mmol/L)	-	-	0.53	-
$CaCl_2*2H_2O$ (mmol/L)	-	2.72	1.36	1.51
HCl (37%) (mL/L)	-	6.50	0.18	0.15
Urea (mmol/L)	3.33	1.42	1.67	4.16
Glucose (mmol/L)	-	3.61	-	-
Glucuronic acid (mmol/L)	-	0.10	-	-
Uric acid (mmol/L)	0.09	-	-	-
Glucoseamine hydrochloride (mmol/L)	-	1.53	-	-
Bovine serum albumin (g/L)	-	1.00	1.00	1.80
Mucin (g/L)	0.025	3.00	-	-
A. oryzae α-amylase (g/L)	0.29	-	-	-
A. niger lipase (U/mL)	-	100	-	-
Pepsin (g/L)	-	2.50	-	-
Pancreatin (g/L)	-	-	9.00	-
Lipase type II from porcine pancreas (g/L)	-	-	1.50	-
Bovine bile extract (g/L)	-	-	-	18.75
**pH**	6.8±0.0	1.4±0.1	8.06±0.0	8.1±0.1

Table S2. Chemical shift assignments and multiplicities of the ^{1}H NMR signals in $CDCl_3$ of protons of glycerides. TG: triglycerides; DG: diglycerides; MG: monoglycerides. The signal letters agree with those given in Figure 1.

Signal	Chemical shift (ppm)	Multiplicity	Type of protons	Structures
			**Glycerides structure protons**	
**I**	3.65	ddd	ROCH₂–CHOH–C**H̲**₂OH	glyceryl group in **1-MG**
**J**	3.73	m*	ROCH₂–CH(O*R*')–C**H̲**₂OH	glyceryl group in **1,2-DG**
**K**	3.84	m*	HOC**H̲**₂–CH(O*R*)–C**H̲**₂OH	glyceryl group in **2-MG**
**L**	3.94	m	ROCH₂–C**H̲**OH–CH₂OH	glyceryl group in **1-MG**
**M**	4.05-4.21	m	ROC**H̲**₂–C**H**OH–C**H̲**₂OR'	glyceryl group in **1,3-DG**
**N**	4.18	ddd	ROC**H̲**₂–CHOH–CH₂OH	glyceryl group in **1-MG**
**O**	4.22	dd,dd	ROC**H̲**₂–CH(O*R*')–C**H̲**₂OR''	glyceryl group in **TG**
**P**	4.28	ddd	ROC**H̲**₂–CH(O*R*')–CH₂OH	glyceryl group in **1,2-DG**
**Q**	4.93	m	HOCH₂–C**H̲**(O*R*)–CH₂OH	glyceryl group in **2-MG**
**R**	5.08	m	ROCH₂–C**H̲**(O*R*')–CH₂OH	glyceryl group in **1,2-DG**

Abbreviations: d: doublet; m: multiplet.

*This signal shows different multiplicity if the spectrum, is acquired from the pure compound or taking part in the mixture.

**The intensity of some of these signals, also shown in Figure 1, together with signal F of Table S3, were used to estimate the molar percentages of different kinds of glyceryl structures using the equations [eq. S1-eq. S10].

***The assignment of the ^{1}H NMR signals of the protons was made as in previous studies (Guillén & Uriarte, 2012; Nieva-Echevarría et al., 2014).

Table S3. Chemical shift assignments and multiplicities of the ^{1}H NMR signals in $CDCl_3$ of protons of acyl groups and fatty acids. AG: acyl groups; FA: fatty acids. The signal letters agree with those given in Figure 1.

Signal	Chemical shift (ppm)	Multiplicity	Type of protons	Structures
			**Main acyl groups (AG) and fatty acids (FA)**	
A	0.88	t	–C**<u>H</u>**$_3$	saturated and monounsaturated **ω-9** in **AG** and **FA**
	0.89	t	–C**<u>H</u>**$_3$	linoleic in **AG** and **FA**
B	0.97	t	–C**<u>H</u>**$_3$	linolenic in **AG** and **FA**
C	1.19–1.42	m**	–(C**<u>H</u>**$_2$)$_n$–	**AG** and **FA**
D	1.61	m	–OCO–CH$_2$–C**<u>H</u>**$_2$–	**AG** in **TG**
	1.62	m	–OCO–CH$_2$–C**<u>H</u>**$_2$–	**AG** in **1,2-DG**
	1.63	m	–OCO–CH$_2$–C**<u>H</u>**$_2$–, COOH–CH$_2$–C**<u>H</u>**$_2$–	**AG** in **1,3-DG**, **1-MG** and **FA**
	1.64	m	–OCO–CH$_2$–C**<u>H</u>**$_2$–	**AG** in **2-MG**
E	1.92–2.15	m***	–C**<u>H</u>**$_2$–CH=CH–	**AG** and **FA**
F*	2.26–2.36	dt	–OCO–C**<u>H</u>**$_2$–	**AG** in **TG**
	2.33	m	–OCO–C**<u>H</u>**$_2$–	**AG** in **1,2-DG**
	2.35	t	–OCO–C**<u>H</u>**$_2$–, COOH–C**<u>H</u>**$_2$–	**AG** in **1,3-DG**, **1-MG** and **FA**
	2.38	t	–OCO–C**<u>H</u>**$_2$–	**AG** in **2-MG**
G	2.77	t	=HC–C**<u>H</u>**$_2$–CH=	linoleic in **AG** and **FA**
H*	2.80	t	=HC–C**<u>H</u>**$_2$–CH=	linolenic in **AG** and **FA**

Abbreviations: d: doublet; t: triplet; m: multiplet.
*The intensity of these signals, also shown in Figure 1, was used to estimate the molar percentage of linolenic acyl groups plus fatty acids by using equations [eq. S11].
**Overlapping of multiplets of methylenic protons in the different acyl groups either in β-position, or further, in relation to double bonds, or in γ-position, or further, in relation to the carbonyl group.
***Overlapping of multiplets of the α-methylenic protons in relation to a single double bond of the different unsaturated acyl groups.
****The assignment of the ^{1}H NMR signals of the protons was made as in previous studies (Guillén & Ruiz, 2003; Nieva-Echevarría et al., 2014).

Table S4. Chemical shift assignments and multiplicities of the ^{1}H NMR signals in $CDCl_3$ of protons of some oxidation compounds detected in the digestates and formed during the *in vitro* digestion.

Signal	Chemical shift (ppm)	Multiplicity	Type of protons	Structures
			**Oxidation Compounds (OC)**	
**Conjugated dienic systems associated with hydroperoxy groups**				
**-**	5.51	dtm	–C**H**=CH–CH=CH–	(*Z,E*)-conjugated double bonds associated with hydroperoxy group (OOH) in octadecatrienoic **AG** and **FA** **HPO-c(*Z,E*)-dEs**
**-**	5.56	ddm		
**-**	6.00	ddtd		
**b**	***6.58***	dddd		
**Aldehydes**				
**f**	***9.75***	t	-C**H**O	**n-alkanals**
	2.40	dt	$-CH_2-$	

Abbreviations: d: doublet; t: triplet; m: multiplet.

*The intensities of the signals indicated in bold, together with signal D of Table S3, were used to estimate the concentration (mmol/molAG+FA) using the equation [eq. S12].

**The assignment of the ^{1}H NMR signals of the protons was made with the aid of standard compounds and with the data taken from literature (Guillén & Ruiz, 2005).

Table S5. Chemical shift assignments and multiplicities of the ^{1}H NMR signals in $CDCl_3$ of protons of cycloartenol and methylencycloartenol, esters of cycloartenol and methylencycloartenol, *gamma*-tocopherols, hydroxytyrosol acetate and dodecyl gallate detected in the samples before and after *in vitro* digestion. Some of the signal letters agree with those given in Figure 2.

Signal	Chemical shift (ppm)	Multiplicity	Type of protons	Compounds
		**Sterols**		
**4,4´-DiMe-St.**	***0.33***[**]	d	-C**H**$_2$- (exo, C-19)	Cycloartenol/ 24-Methylenecycloartenol
**4,4´-DiMe-St´.**	***0.34***[**]	d	-C**H**$_2$- (exo, C-19)	Esters of Cycloartenol/ 24-Methylenecycloartenol
		***gamma*-tocopherol**		
**γT**	***6.36***[**]	s	-C**H** (C-5)	*gamma*-tocopherol
		**Hydroxytyrosol acetate**		
	6.60	dd	Ar**H** (C-8)	
**HTA**	***6.75***[**]	d	Ar**H** (C-4)	Hydroxytyrosol acetate
	6.78	d	Ar**H** (C-7)	
		**Dodecyl gallate**		
**DG**	***7.20***	s	Ar**H**(C-3; C-7)	Dodecyl gallate

Abbreviation: s: singlet; d: doublet.

*The intensity of these signals, together with signal D of Table S3, were used to estimate the concentration (mmol/molAG+FA) using the equation [eq. S12].

**Assignment was made with the aid of standard compounds and with the data taken from the literature (Baker & Mayers, 1991; Kubo et al., 2002; Kawai et al., 2007)

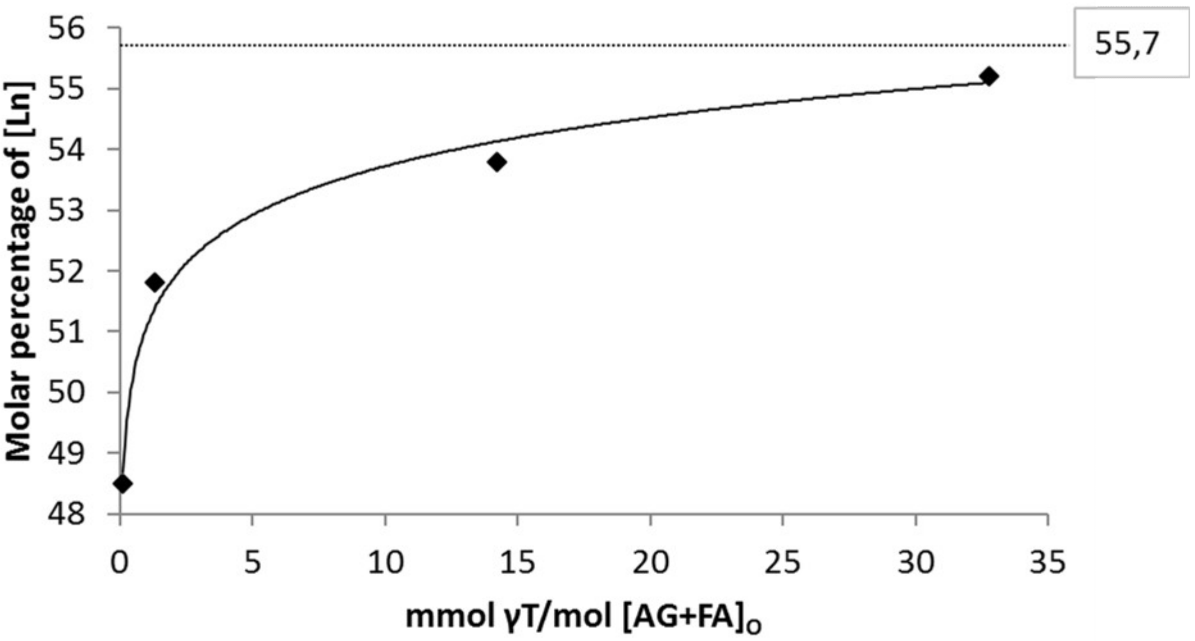

Figure S1. Graphical representation of linolenic structures concentration in the digestates of the different virgin flaxseed oil samples enriched in *gamma*-tocopherol given in molar percentage of [Ln] referred to the total moles of $[AG+FA]_D$ *versus* enrichment level of *gamma*-tocopherol in the corresponding oil samples, given in mmol $\gamma T/mol[AG+FA]_O$.

Quantification from ^{1}H NMR spectral data of several compounds present in the starting samples and/or in the lipid extracts of the digestates.

Bearing in mind that the area of each ^{1}H NMR spectral signal is proportional to the number of protons that generate it, and that the proportionality constant is the same for all kinds of protons, the area of some spectral signals can be employed to quantify a wide variety of compounds, as detailed below.

A. Equations used to estimate the molar percentage (%) of the several glyceride structures present in the lipid extract of digestates and the glycerol.

In these equations, the number of moles (N) of fatty acids and all the glycerides were expressed as follows:

$N_{2\text{-}MG}=Pc*A_K/4$ [eq. S1]

$N_{1\text{-}MG}=Pc*A_L$ [eq. S2]

$N_{1,2\text{-}DG}=Pc*(A_{I+J}-2A_L)/2$ [eq. S3]

$N_{TG}=Pc*(2A_{4.26\text{-}4.38}-A_{I+J}+2A_L)/4$ [eq. S4]

$N_{1,3\text{-}DG}=Pc*(A_{4.04\text{-}4.38}-2A_{4.26\text{-}4.38}-2A_L)/5$ [eq. S5]

$N_{FA}=(Pc*A_F-6N_{TG}-4N_{1,2\text{-}DG}-4N_{1,3\text{-}DG}-2N_{1\text{-}MG}-2N_{2\text{-}MG})/2$ [eq. S6]

$N_{Gol}=(N_{FA}-N_{1,2\text{-}DG}-N_{1,3\text{-}DG}-2N_{2\text{-}MG}-2N_{1\text{-}MG})/3$ [eq. S7]

where Pc is the proportionality constant existing between the area of the ^{1}H NMR signals and the number of protons that generate them, A_K, A_L, A_{I+J} and A_F are the areas of the corresponding signals indicated in Table S2, and $A_{4.26\text{-}4.38}$ and $A_{4.04\text{-}4.38}$ represent the areas of the signals between 4.26 and 4.38 ppm, and between 4.04 and 4.38 ppm, respectively.

Using these equations, the molar percentages of the different kinds of glycerides in relation to the total number of moles of glyceryl structures present (NT_{GS}) were determined as follows:

$NT_{GS}=N_{TG}+N_{1,2\text{-}DG}+N_{1,3\text{-}DG}+N_{2\text{-}MG}+N_{1\text{-}MG}+N_{Gol}$ [eq. S8]

$G\%=100N_G/NT_{GS}$ [eq. S9]

where G is each kind of glyceride (TG, 1,2-DG, 1,3-DG, 2-MG and 1-MG) and N_G the respective number of moles.

$Gol\%=100N_{Gol}/NT_{GS}$ [eq. S10]

B. Estimation of the molar percentages of linolenic fatty acids (FA) plus acyl groups (AG) (FA+AG). The molar percentages of linolenic (Ln%) FA plus AG, in

relation to the total number of moles of AG plus FA (NT_{AG+FA}) present in the starting oils and in the lipid extracts of the corresponding digestates were estimated as follows:

$$Ln\% = 100*(A_H/2*A_F) \quad \text{[eq. S11]}$$

where A_H and A_F are the areas of signals H and F indicated in Table S3.

C. Estimation of the concentration of specific compounds in oil samples and in the lipids extract from digestates.

The concentration of the several kinds of specific compounds (SC), expressed as micromoles per mole of the sum of AG+FA present, was estimated by using the following equations:

$$[SC] = [(A_{SC}/n)/(A_D/2)]*1000 \quad \text{[eq. S12]}$$

where A_{SC}, is the areas of the signals selected for the quantification of each specific compound (SC), present in the oil samples and in the lipid extract from digestates and n the number of protons that generate each signal given in Tables S4 and S5 and Figure 1 and A_D is the area of the signal D in Table S3.

REFERENCES

Baker, J.K.; Myers, C.W. One-dimensional and two-dimensional ^{1}H-and ^{13}C-nuclear magnetic resonance (NMR) analysis of vitamin E raw materials or analytical reference standards. *Pharm. Res.* **1991**, *8*(6), 763-770.

Guillén, M.D.; Ruiz, A. Rapid simultaneous determination by proton NMR of unsaturation and composition of acyl groups in vegetable oils. *Eur. J. Lipid Sci. Technol* **2003**, *105*(11), 688-696.

Guillén, M.D.; Ruiz, A. Oxidation process of oils with high content of linoleic acyl groups and formation of toxic hydroperoxy-and hydroxyalkenals. A study by ^{1}H nuclear magnetic resonance. *J. Sci. Food Agric* **2005**, *85*(14), 2413-2420.

Guillén, M.D.; Uriarte, P.S. Study by ^{1}H NMR spectroscopy of the evolution of extra virgin olive oil composition submitted to frying temperature in an industrial fryer for a prolonged period of time. *Food Chem.* **2012**, *134*(1), 162-172.

Kawai, S.; Takada, Y.; Tsuchida, S.; Kado, R.; Kimura, J. Sterols from bivalves *Calyptogena soyoae* and *Bathymodiolus septemdierum* living in deep sea. *Fish. Res.* **2007**, *73*(4), 902-906.

Kubo, I., Xiao, P., Nihei, K. I., Fujita, K. I., Yamagiwa, Y., & Kamikawa, T. Molecular design of antifungal agents. *J. Agric. Food Chem* **2002**, *50*(14), 3992-3998.

Nieva-Echevarría, B.; Goicoechea, E.; Manzanos, M.J.; Guillén, M.D. A method based on ^{1}H NMR spectral data useful to evaluate the hydrolysis level in complex lipid mixtures. *Food Res Int* **2014**, *66*, 379-387.

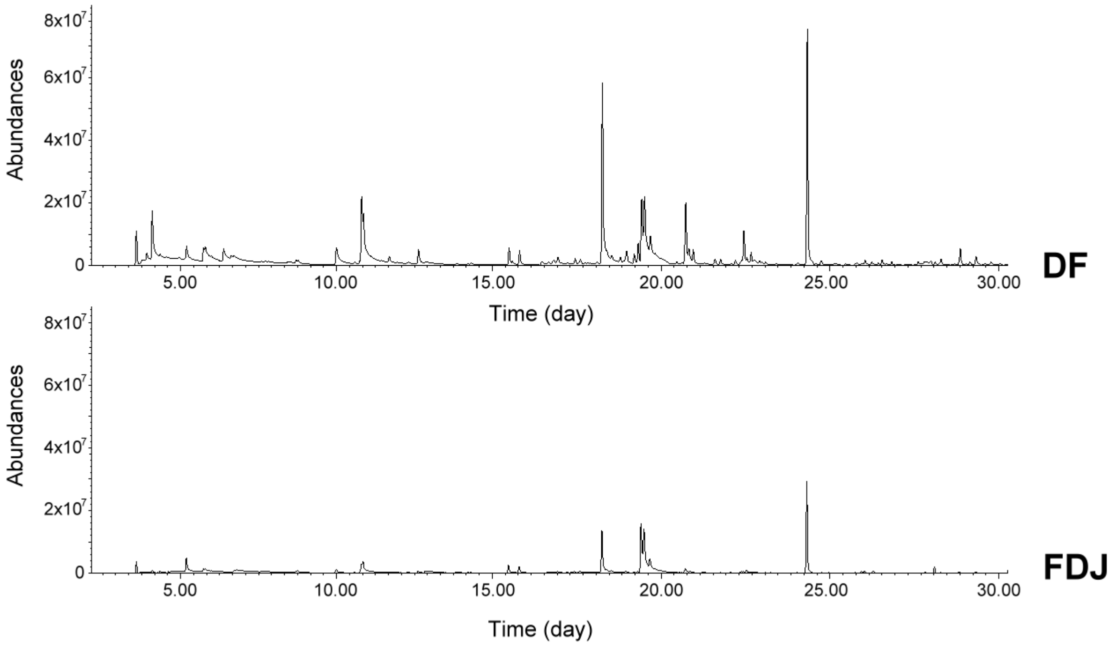

Figure S2. Region between 4-30 min of the total ion chromatogram obtained by SPME-GC/MS of the FDJ sample and of the digestate of the virgin flaxseed oil DF.
